# Astaxanthin Alleviates the Decline of Sperm Quality Caused by Heat Stress in Mice via Reducing Oxidative Stress

**DOI:** 10.3390/life15060851

**Published:** 2025-05-25

**Authors:** Jing Wang, Yuchuan Luo, Yifeilong He, Wanzhen Li, Yinghe Qin, Yingjie Wu

**Affiliations:** 1 State Key Laboratory of Animal Nutrition and Feeding, College of Animal Science and Technology, China Agricultural University, Beijing 100193, China; w13375541018@163.com (J.W.); lyuchuan2022@163.com (Y.L.); heyifeilong@163.com (Y.H.); lwzhen2022@163.com (W.L.); 2 National Engineering Laboratory for Animal Breeding, College of Animal Science and Technology, China Agricultural University, Beijing 100193, China

**Keywords:** heat stress, astaxanthin, oxidative stress, sperm motility and viability

## Abstract

Heat stress impairs spermatogenesis primarily through increased testicular oxidative stress. Astaxanthin, a potent antioxidant, has shown beneficial effects in sperm cryopreservation. However, its potential to mitigate testicular damage induced by elevated temperatures remains uninvestigated. In this study, male mice were administered astaxanthin (10 mg/kg/d, 50 mg/kg/d, and 100 mg/kg/d) via gavage for 21 days, with heat exposure occurring during the final 14 days. Samples were collected after the last treatment or following a recovery period. Spermatozoa in the cauda epididymis were assessed using computer-aided sperm analysis (CASA) or Diff-Quik staining, and serum testosterone levels and oxidative stress markers in both serum and testis were quantified via enzyme-linked immunosorbent assay (ELISA). Heat treatment resulted in significant reductions in sperm motility, viability, and morphological integrity. However, daily supplementation of astaxanthin at 50 mg/kg and 100 mg/kg effectively alleviated these heat-induced impairments. Furthermore, astaxanthin at 50 mg/kg/d notably improved testis weight, testis index, and serum testosterone levels under heat stress. Its antioxidant capacity was validated by significant restoration of total antioxidant capacity (T-AOC), superoxide dismutase (SOD), and reduction in malondialdehyde (MDA) levels in both testis and serum. In conclusion, this study highlights the protective effects of astaxanthin against heat-induced testicular and sperm damage by reducing oxidative stress, supporting its potential use as a nutritional or nutraceutical supplement to promote male reproductive health, particularly in the context of summer subfertility in farm animals.

## 1. Introduction

Infertility affects 10–15% of reproductive-aged couples and presents significant clinical, social, and economic challenges [1], with male factors contributing to 40–50% of cases [2,3]. Testicular temperature is recognized as a key factor in male infertility by the World Health Organization, given the strong correlation between elevated testicular temperature and reduced fertility [2,4]. Positioned outside the body in most adult animals, the testis is particularly vulnerable to heat stress, as it must be maintained 2–7 °C below core body temperature to support normal spermatogenesis [5]. Various external and internal factors, such as hot baths, saunas, prolonged occupational heat exposure (e.g., chefs, welders), varicocele, cryptorchidism, and fever, can raise testicular temperature and contribute to testicular heat stress [6,7,8]. This heat stress induces testicular damage, impairing sperm motility, viability, and morphology, which significantly increases the risk of infertility.

Oxidative stress plays a central role in heat stress-induced testicular injury, resulting from mitochondrial dysfunction, apoptosis, autophagy, DNA damage, and disruption of the blood/testis barrier (BTB), among other factors [7]. Budzinska et al. demonstrated that prolonged scrotal hyperthermia reduces sperm quality in humans by directly triggering oxidative stress, affecting plasma membrane fluidity, mitochondrial stability, and sperm DNA integrity [9]. Spermatozoa are particularly vulnerable to lipid peroxidation due to their high content of polyunsaturated fatty acids [10]. Additionally, electrophilic aldehydes such as 4HNE and acrolein, generated by human sperm mitochondria, are potent inducers of apoptosis, lipid peroxidation, and DNA damage [11].

Astaxanthin, a natural antioxidant, possesses robust antioxidant properties and is considered safe for use [12]. It exhibits a wide array of biological activities, including anti-apoptotic, anti-inflammatory, antioxidative, and neuroprotective effects [13]. The molecule’s unique structure, particularly the central polyene chain with thirteen conjugated double bonds, enables it to neutralize free radicals and donate electrons, contributing to its powerful antioxidant capacity [14]. In male reproduction, astaxanthin has been shown to protect sperm during the freezing/thawing process in humans [15], boars [16], and roosters [17]. Furthermore, it enhances testosterone synthesis in aging roosters and supports human sperm capacitation [18,19]. However, the potential mechanisms by which astaxanthin mitigates sperm damage in high-temperature environments remain unclear.

This study investigates the protective effects of astaxanthin on sperm quality under heat stress and its antioxidative properties in the testis. The findings will not only support the application of astaxanthin in male reproductive health but also advance research on animal fertility and infertility.

## 2. Materials and Methods

### 2.1. Ethics Approval

This study was approved by the Institutional Ethics Committee of China Agricultural University. All experiments were conducted in strict adherence to the Guidelines of Beijing Municipality on the Review of Welfare and Ethics of Laboratory Animals and The Guideline on the Humane Treatment of Laboratory Animals (MOST 2006a).

### 2.2. Animals and Experimental Design

Eight-week-old male ICR mice (35 ± 5 g) were obtained from Spiff Biotechnology Co., Ltd. (Beijing, China). The mice were housed in a controlled environment with a temperature of 20–25 °C, relative humidity of 50–70%, and a 12-h light/dark cycle, with ad libitum access to food and water.

Experiment 1: A total of 75 mice were randomly divided into five groups (15 mice per group) and exposed to heat at 37 °C for 0 h (control), 1 h, 2 h, 3 h, or 4 h per day in an incubator. The heat exposure lasted for 14 consecutive days, which corresponds to the developmental period for heat-stress-sensitive spermatocytes to mature into intact spermatozoa (approximately 13 days in mice) [20,21]. Five mice from each group were sacrificed immediately after the final heat exposure, while the remaining mice were allowed to recover at normal temperature (20–25 °C). Five mice per group were sacrificed after one week, and five were sacrificed after two weeks to assess recovery. The recovery groups were established to evaluate heat stress-induced damage to spermatozoa (or precursor spermatogenic cells) in the testes by assessing sperm motility and viability in the cauda epididymis. This was because it takes approximately 10 days for murine spermatozoa to travel from the seminiferous tubules to the epididymis [22]. One epididymis from each mouse was isolated for sperm motility analysis.

Experiment 2: A total of 50 mice were randomly divided into five groups (10 mice per group). Mice in the control (CON) and heat stress (HS) groups received daily intragastric administration of 5 mL/kg corn oil, while mice in the astaxanthin treatment groups (HS+AST-L, HS+AST-M, and HS+AST-H) received daily intragastric doses of 10 mg/kg, 50 mg/kg, and 100 mg/kg astaxanthin, respectively.

**Figure 1:**
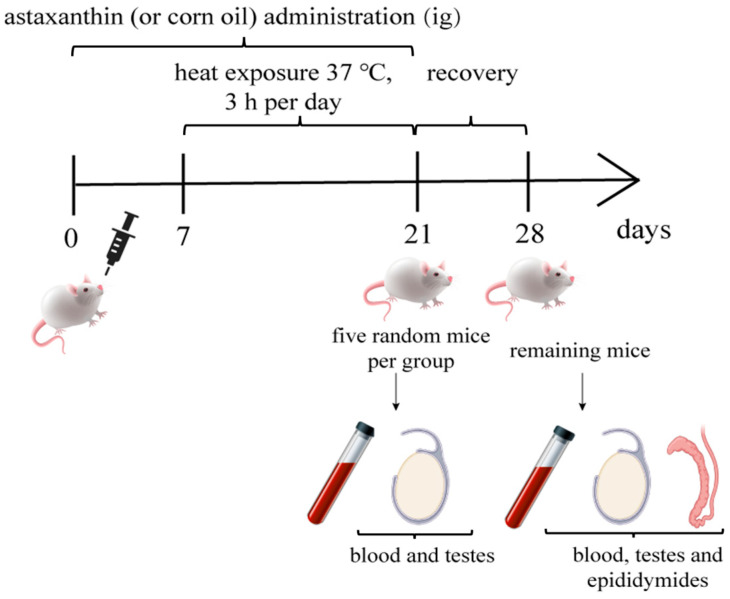
Basic flow chart of Experiment 2.

Astaxanthin (or corn oil) administration continued for 21 days (Figure 1). Starting on day 7, mice in the HS and HS+AST groups were subjected to 37 °C heat exposure for 3 h per day (Figure 1). Mice were weighed twice a week. Five mice from each group were sacrificed for blood and testes collection immediately after the final heat treatment, and the remaining mice were sacrificed for blood, testes, and epididymides after a one-week recovery (Figure 1).

### 2.3. Analysis of Sperm Motility, Viability, and Morphology

Sperm motility, viability, and morphology were assessed following previously described protocols in our laboratory [19,20,21,22,23,24]. Briefly, the cauda epididymis was isolated from the mouse and divided into small pieces. Sperm were allowed to swim out in culture at 37 °C for 15 min. Sperm parameters were analyzed using computer-aided sperm analysis (CASA) with a Zeiss Axioscope A1 microscope (Carl Zeiss AG, Oberkochen, Germany) and LINE analysis software (Version 5.0). For morphological analysis, sperm smears were prepared and stained using Diff-Quik, following the protocol provided by Beijing Leagene Biotechnology Co. (Beijing, China). At least 200 spermatozoa per mouse were counted in randomly selected high-power fields, following the sperm morphological standard.

### 2.4. Determination of Testosterone Level in Serum

Blood samples were collected from anesthetized mice (five per group) via retro-orbital sinus puncture using a capillary tube. After coagulation, blood was centrifuged at 3000× *g* at 4 °C for 20 min. The serum was separated, transferred to a new tube, and stored at −80 °C until further analysis.

Serum testosterone levels were determined using a specific enzyme-linked immunosorbent assay (ELISA) kit from the Shanghai Yuanjie Biotechnology Center (Shanghai, China). The assay was conducted according to the manufacturer’s instructions. The sensitivity of the assay was 0.05 ng/mL, with intra-assay coefficients of variation (CV) < 10% and inter-assay CV < 15%. Data were expressed as nanograms per milliliter of serum.

### 2.5. Determination of Oxidative Indexes Level in Testis and Serum

Testes from five mice per group were homogenized in ice-cold PBS (0.1 g testis/mL PBS). The homogenate was centrifuged at 1200× *g* at 4 °C for 10 min, and the supernatant was collected. Levels of total antioxidant capacity (T-AOC), superoxide dismutase (SOD), and malondialdehyde (MDA) in both testicular supernatant and serum were measured using ELISA kits (Beyotime Biotechnology, Beijing, China) according to the manufacturer’s instructions.

### 2.6. Statistical Analysis

Data were presented as mean ± SEM. One-way ANOVA (GraphPad, version 5.0) followed by post hoc Tukey’s test was used to compare group differences. A *p*-value of <0.05 was considered statistically significant.

## 3. Results

### 3.1. Heat Stress Decreases Sperm Motility with a Carryover Effect

**Figure 2:**
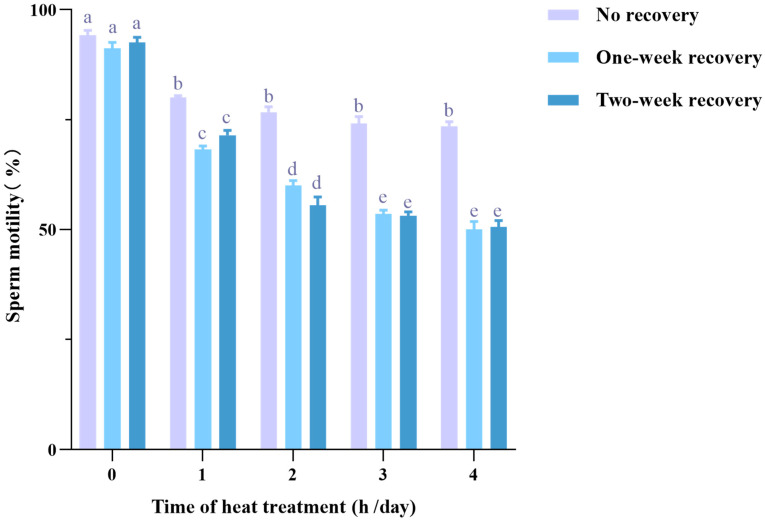
Heat stress decreases sperm motility in mice with a carryover effect. ICR mice (5 mice per group) were subjected to varying heat treatments (0 to 4 h per day) for 14 consecutive days, followed by different recovery durations (no recovery, one week, and two weeks). Sperm motility was measured in each group using CASA. Lowercase letters indicate significant differences between groups (*p* < 0.05).

To evaluate the detrimental effects of heat stress on sperm quality, mice were exposed to 37 °C for varying durations (0 to 4 h) per day over a two-week period. Subsequently, spermatozoa were extracted from the cauda epididymis for motility assessment after different recovery durations. As shown in Figure 2, in the no-recovery groups, sperm motility was initially 94.22 ± 1.11%, but heat treatments of 1 h, 2 h, 3 h, and 4 h per day led to a reduction in motility to a similar extent, ranging from 80.04 ± 0.43% to 73.55 ± 0.96%. Notably, when sampling was delayed to one or two weeks after heat treatment (recovery), sperm motility decreased even further. The most significant decline, to 50.03 ± 1.81%, was observed in the 4-h/day heat treatment group with a one-week recovery. Additionally, in both the one-week and two-week recovery groups, sperm motility showed a negative correlation with the duration of heat treatment, but there was no further decline in motility between the 3-h/day and 4-h/day groups. The differences in sperm motility between the one-week and two-week recovery groups were minimal. These findings confirm that heat stress significantly reduces sperm motility with a lasting effect. Consequently, the following experimental protocol was adopted, exposing mice to 37 °C for 3 h per day over 14 consecutive days to impose heat stress.

### 3.2. Astaxanthin Alleviates Heat Stress-Induced Decline of Sperm Quality

**Figure 3:**
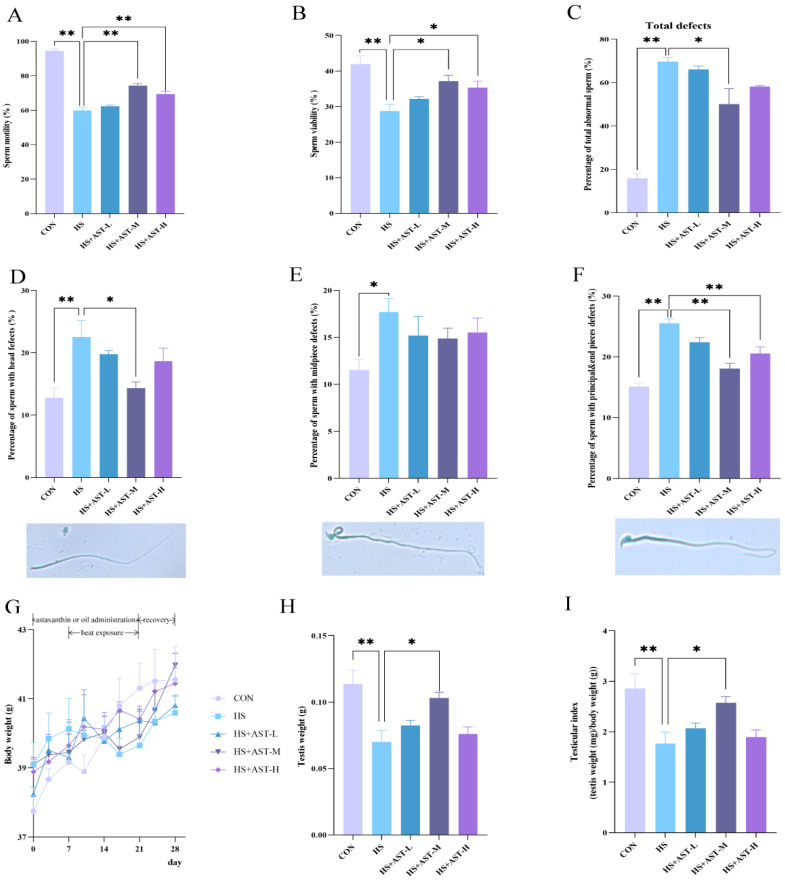
Astaxanthin ameliorates heat stress-induced impairments in sperm quality and testicular morphometric parameters. Mice were subjected to heat treatment (3 h per day for 14 days) with or without astaxanthin supplementation (10, 50, or 100 mg/kg). Cauda spermatozoa and testes were collected after one week of recovery. (**A**,**B**) Sperm motility and sperm viability assessed by CASA (5 mice per group). (**C**–**F**) The percentage of morphologically abnormal sperm assessed by Diff-Quik staining, analyzing both total and various types of sperm defects (3 mice per group). (**G**–**I**) Body weight, testis weight, and testis index. Body weights were measured throughout the experiment; testis weights were measured (averaged) post-sampling, and the testis index was calculated as testis weight/body weight (5 mice per group). * *p* < 0.05, ** *p* < 0.01.

To assess the effects of astaxanthin supplementation on heat stress-induced reductions in sperm quality, mice were treated with heat (3 h per day for 14 days) in the presence or absence of astaxanthin (10, 50, or 100 mg/kg). After one week of recovery, sperm motility, viability, and morphology were evaluated. Consistent with prior findings, heat-treated mice showed a significant decrease in sperm motility, which dropped by approximately 35%, from 94.54 ± 1.01% (control) to 59.91 ± 1.66% (Figure 3A). Similarly, sperm viability was reduced from 42.00 ± 2.32% (control) to 28.77 ± 1.88% after heat exposure (Figure 3B). Importantly, medium-dose (50 mg/kg) and high-dose (100 mg/kg) astaxanthin demonstrated significant protective effects against heat stress (*p* < 0.05), but the low-dose (10 mg/kg) did not show a notable protective effect (*p* > 0.05, Figure 3A,B). Morphological analysis using Diff-Quik staining revealed a sharp increase in the percentage of abnormal sperm following heat treatment, from 15.85 ± 1.92% to 69.73 ± 1.82% (Figure 3C). Examination of the types of sperm abnormalities showed that principal and end piece defects, such as double, crooked, or coiled tails, accounted for the largest proportion, followed by head defects like oversized, undersized, or misshapen heads (Figure 3D,F). As expected, medium-dose astaxanthin significantly improved sperm morphology by reducing the overall percentage of sperm defects, as well as defects in the head and principal/end pieces (Figure 3C–F). High-dose astaxanthin also mitigated defects in the principal and end pieces (Figure 3F). These results suggest that astaxanthin alleviates the decline in sperm quality induced by heat stress, with the optimal dosage being approximately 50 mg/kg.

To explore the potential effects of astaxanthin on body and testicular weight, mice were weighed twice a week during the experiment, and both testes were collected and weighed at the end of the study. There were no significant differences in body weight between groups, indicating that neither heat treatment nor astaxanthin supplementation significantly impacted body weight (Figure 3G). However, heat treatment resulted in a marked reduction in testis weight and testis index, which was nearly completely reversed by medium-dose astaxanthin supplementation (Figure 3H,I). These results suggest that astaxanthin effectively alleviates testicular damage caused by heat stress.

### 3.3. Astaxanthin Restores Serum Testosterone Level in Heat-Stressed Mice

**Figure 4:**
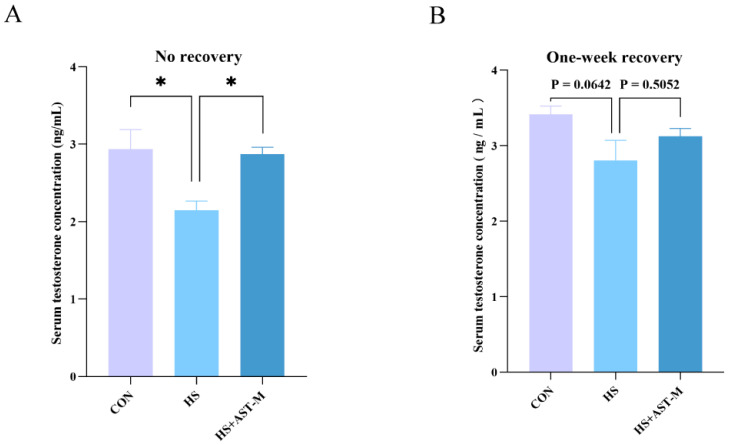
Astaxanthin decreases serum testosterone levels in heat-stressed mice. Serum testosterone concentration was measured using an ELISA assay (5 mice per group). (**A**) Testosterone levels in mice sampled immediately after heat stress. (**B**) Testosterone levels in mice sampled after one week of recovery. * *p* < 0.05.

To examine changes in testosterone levels, serum testosterone concentrations were measured in the control, heat stress, and heat stress + medium-dose astaxanthin groups using ELISA. In the no-recovery group, serum testosterone levels were significantly reduced by heat treatment, but supplementation with astaxanthin restored testosterone levels to near normal (Figure 4A). However, there was no significant difference in testosterone levels among the three groups after one week of recovery, although a similar trend was observed (Figure 4B). These results suggest that astaxanthin helps alleviate androgen deficiency induced by heat stress, and testosterone levels may naturally recover with time following the cessation of heat exposure.

### 3.4. Astaxanthin Reduces Heat Stress-Induced Oxidative Stress

**Figure 5:**
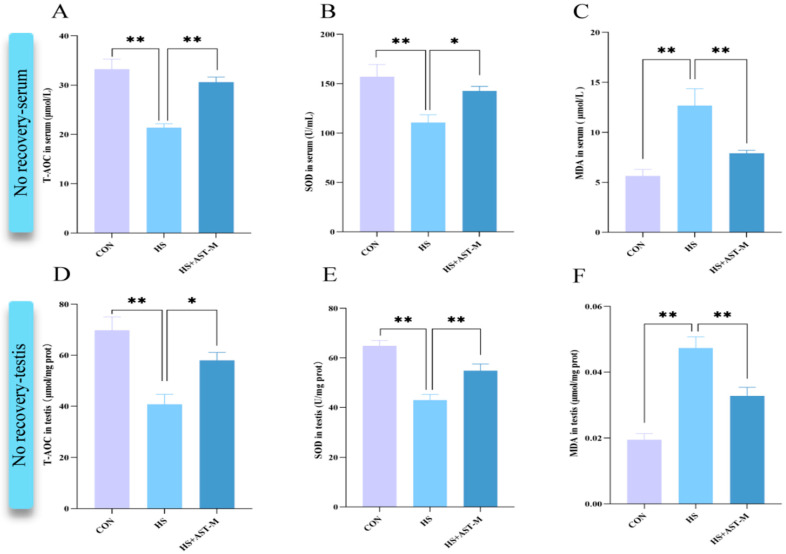

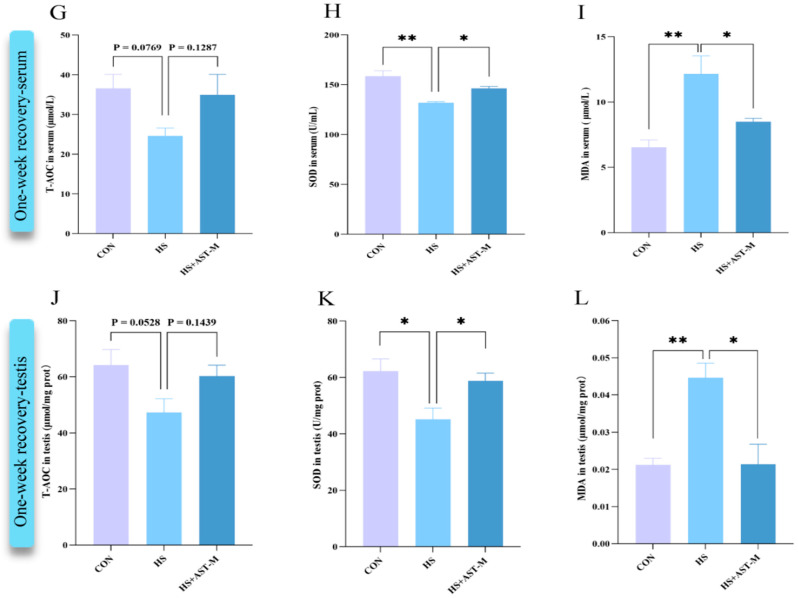
Astaxanthin reduces the levels of oxidative stress in serum and testis of heat-stressed mice. The levels of T-AOC, SOD, and MDA in serum (**A**–**C**,**G**–**I**) and testis (**D**–**F**,**J**–**L**) were measured by ELISA (5 mice per group). (**A**–**F**) No recovery. (**G**–**L**) One-week recovery. * *p* < 0.05, ** *p* < 0.01. Prot: Protein.

It is well-established that heat stress induces oxidative stress, while astaxanthin is a potent antioxidant. To evaluate the antioxidant capacity of astaxanthin in this study, oxidative stress indicators (T-AOC, SOD, and MDA) in the serum and testis of mice sampled were measured immediately after heat treatment or after one week of recovery by ELISA. In the no-recovery group, heat treatment significantly reduced the levels of SOD and T-AOC and increased MDA levels both in serum and testis. Astaxanthin supplementation restored these markers to varying degrees (Figure 5A–F). After one week of recovery, results mirrored those of the no-recovery group. SOD levels decreased in the heat stress group and showed partial restoration in the astaxanthin-supplemented group (Figure 5H,K). MDA levels were elevated in the heat stress group but returned to normal following astaxanthin supplementation (Figure 5I,L). However, T-AOC levels in serum and testis did not show a significant difference among the three one-week recovery groups, although there was a trend towards lower levels in the heat stress group and recovery in the astaxanthin-supplemented group (Figure 5G,J). Additionally, protein immunoblotting of cleaved Caspase-3, a marker of apoptosis, revealed that heat treatment significantly increased cleaved Caspase-3 levels in testicular tissue (*p* < 0.05). Astaxanthin supplementation reversed this effect, suggesting that astaxanthin mitigates heat stress-induced apoptosis of testicular cells, further supporting our conclusions. These data have been included in the Supplementary Material as Figure S1. In summary, these results demonstrate that astaxanthin effectively alleviates oxidative stress in both the testis and serum of mice.

## 4. Discussion

The present study demonstrates that astaxanthin supplementation alleviates the decline in sperm quality in mice induced by heat stress, primarily through the reduction of oxidative stress, thereby revealing a novel role of astaxanthin in male reproduction.

Our findings confirm the detrimental impact of heat stress on sperm quality, as documented in several studies. For instance, Cao et al. showed that heat stress adversely affects the male reproductive system by disrupting the testicular microenvironment, leading to reduced sperm quality [25]; Shahat et al. observed that elevated testicular temperature negatively impacts sperm motility, morphology, and fertility, inducing germ cell apoptosis and DNA damage, with the extent of these effects correlating to the degree and duration of the temperature increase [26]. Additionally, this study substantiates the carryover effect following the cessation of heat treatment. This aligns with our previous research on male rabbits, which demonstrated that sperm count did not return to baseline until eight weeks after a nine-week heat exposure period [27]. This observation is further supported by fertility data in cattle, which indicate that the effects of summer heat stress extend into the autumn months in the Northern Hemisphere [28]. The underlying cause of this carryover effect is the prolonged process of spermatogenesis; spermatocytes or spermatids subjected to thermal stress may carry the damage into subsequent developmental stages.

Our findings also reveal that heat stress significantly decreased serum testosterone levels, consistent with previous studies linking dysfunctional testosterone secretion to the decline in male fertility caused by heat stress [29,30]. Several factors contribute to the suppression of testosterone. First, heat stress induces apoptosis in Leydig cells, the primary source of testosterone in the testes [31]. Second, heat stress downregulates key enzymes involved in testosterone biosynthesis, including cytochrome P450 family 17 and steroidogenic acute regulatory protein [32], thereby diverting cholesterol towards cortisol synthesis. Third, the elevated levels of adrenocorticotropic hormone and cortisol induced by heat stress inhibit the secretion of GnRH and LH, ultimately leading to decreased testosterone production [33]. Androgens mediate a wide array of physiological responses and developmental processes through androgen receptor signaling, including the maintenance of spermatogenesis. In the present study, a significant reduction in testicular weight and testicular index was also observed, likely due to severe tissue dehydration and extensive cell apoptosis induced by heat stress. This finding is in line with previous research, which demonstrated that testicular weight decreased to 68.45% after just 25 min of water bath at 43 °C on day 7, suggesting that heat stress may regulate testicular atrophy and spermatogenesis by affecting meiosis and the cell cycle [34]. Furthermore, heat stress is widely known to induce apoptosis in testicular cells, including Leydig cells [35], Sertoli cells [36], and spermatogenic cells [37]. The three principal apoptotic pathways—extrinsic, intrinsic, and endoplasmic reticulum (ER) stress-related—can independently or synergistically induce apoptosis. Notably, heat stress predominantly activates the intrinsic apoptotic pathway, also known as the mitochondrial apoptotic pathway, in spermatocytes and spermatids [38,39]. During this process, cytochrome c is released from the mitochondria into the cytosol [40], where it interacts with apoptotic protease activating factor-1 (Apaf-1) in the presence of ATP/dATP. This interaction leads to the oligomerization of Apaf-1, forming the apoptosome, a multi-protein complex that binds and activates Caspase-9. The activation of Caspase-9 then initiates a cascade of downstream caspase activations, including Caspases-3, -6, and -7, culminating in programmed cell death or apoptosis [41]. Additionally, members of the B-cell lymphoma/leukemia-2 (Bcl-2) protein family play essential roles in regulating mitochondrial membrane permeability [42], and activation of the p53 and p38 mitogen-activated protein kinase (MAPK) signaling pathways contributes to mitochondria-mediated germ cell apoptosis [43].

Astaxanthin possesses remarkable antioxidant capacity, effectively inhibiting oxidative damage and protecting cells from a wide range of pathological conditions [44]. Its unique molecular structure, featuring hydroxyl and keto moieties on each ionone ring [45], allows it to trap radicals in the cell membrane through its polyene chain, while the terminal ring scavenges radicals at both the inner and outer parts of the cell membrane. Consequently, astaxanthin exhibits a particularly high antioxidant activity, which is ten times greater than that of zeaxanthin, lutein, canthaxanthin, and β-carotene, and 100 times higher than α-tocopherol [46]. In the present study, astaxanthin nearly completely alleviates oxidative stress in the testis and serum under high-temperature conditions, as evidenced by the levels of T-AOC, SOD, and MDA. T-AOC reflects the overall antioxidant capacity of the body, SOD activity indicates the strength of the antioxidant system, and MDA content reflects the level of lipid peroxidation. These oxidative stress indicators showed a recovery trend in line with improvements in sperm motility and viability due to astaxanthin supplementation, demonstrating its protective effects against heat-induced damage through the attenuation of oxidative stress.

The dosages of astaxanthin used in this study were based on the previous literature, where the most commonly administered doses for mice ranged from 10 to 120 mg/kg/day. For example, Chen et al. reported that 30 mg/kg/day of astaxanthin for 20 days effectively inhibited oxidative stress, improved liver function, and alleviated gestational diabetes in female mice [47]. Hao et al. found that 50 and 100 mg/kg/day of astaxanthin administered for 3 weeks could regulate serum cytokine levels and improve sperm quality in diabetic KKAy mice [48]. These doses are likely higher than the human-recommended dose of 2–24 mg/day [49], which may be due to differences in bioavailability between species. A study has indicated that the bioavailability of astaxanthin in humans may be 5 to 10 times greater than in mice [50]. This difference in bioavailability is primarily attributed to (1) species-specific variations in gastrointestinal pH, intestinal epithelial tight junctions, bile salt content, and the expression of metabolic enzymes and transporters [51]; and (2) the use of delivery systems in clinical settings, as appropriate oral drug encapsulation methods and formulations can significantly enhance bioavailability, potentially increasing it by up to 18-fold [52]. While clinical medications, including human-use astaxanthin, typically use well-optimized delivery strategies, the animal study did not employ such methods. Consequently, higher doses are often necessary in animal studies. Our findings further support that a dose of 50 mg/kg/day, rather than the lower dose of 10 mg/kg/day, has a significant protective effect against heat stress.

As expected, astaxanthin mitigated the detrimental effects of heat stress on sperm motility and viability. However, its impact appears moderate and limited, as sperm motility and viability in the astaxanthin + heat stress group remained significantly lower than in the control group. Given the effective recovery of oxidative stress markers by astaxanthin, these results suggest that the adverse effects of heat stress on sperm quality are likely not confined to oxidative damage alone [53]. Previous studies have shown that osmotic stress also arises during heat shock [54]. In other words, the molecular response to external heat stress is likely the result of a complex interplay of multiple signaling pathways. In this study, astaxanthin supplementation may have primarily inhibited the oxidative stress pathway, thereby reducing cellular damage, but other contributing factors should also be considered. The mechanisms through which heat stress impairs male reproductive function remain largely unknown. Understanding the biology and mechanisms by which heat stress compromises reproductive performance in male farm animals is essential not only for developing future strategies to mitigate widespread infertility but also for creating effective approaches to address fertility challenges in farm animals during the summer months. Further research is warranted to elucidate these mechanisms.

## 5. Conclusions

Astaxanthin improves sperm motility, viability, and morphology in mice while also restoring testosterone levels under high-temperature conditions. These effects are likely closely linked to its ability to mitigate oxidative stress. These findings suggest that astaxanthin supplementation could serve as a protective intervention for male reproductive health, particularly for individuals exposed to occupational heat stress. Furthermore, it may represent a viable nutritional strategy to reduce heat stress-induced oxidative damage in the reproductive systems of livestock, helping to address issues of summer subfertility.

## Data Availability

Data are contained within the article.

## Supplementary Materials

The following supporting information can be downloaded at: https://www.mdpi.com/article/10.3390/life15060851/s1, Figure S1: Astaxanthin reduces testicular cell apoptosis induced by heat stress.
