# Supplementary material for: Astaxanthin Alleviates the Decline of Sperm Quality Caused by Heat Stress in Mice via Reducing Oxidative Stress
Source: Life (Basel). 2025 May 25;15(6):851. doi: 10.3390/life15060851 (PMC12194212; doi:10.3390/life15060851)
Supplement: Supplementary file 1 [file life-15-00851-s001.zip › life-3525455-supplementary.pdf]

Supplementary Materials: Astaxanthin reduces testicular cell apoptosis induced by heat stress.
(Original images attached at the end)

Figure S1

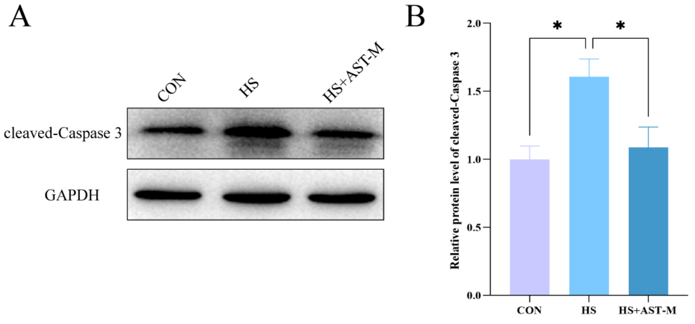

Figure S1 Astaxanthin reduces testicular cell apoptosis induced by heat stress. (A) Immunoblotting of cleaved-Caspase 3 in mouse testes. (B) Gray-scale analysis of the protein. The levels of cleaved-Caspase 3 proteins were represented by their gray ratio to GAPDH and normalized the data for processing (n = 3). *P < 0.05. CON: Control group, HS: Heat treatment group, HS+AST-M: Heat stress + medium-dose astaxanthin group.

Original and Uncropped Images with Densitometric Analysis

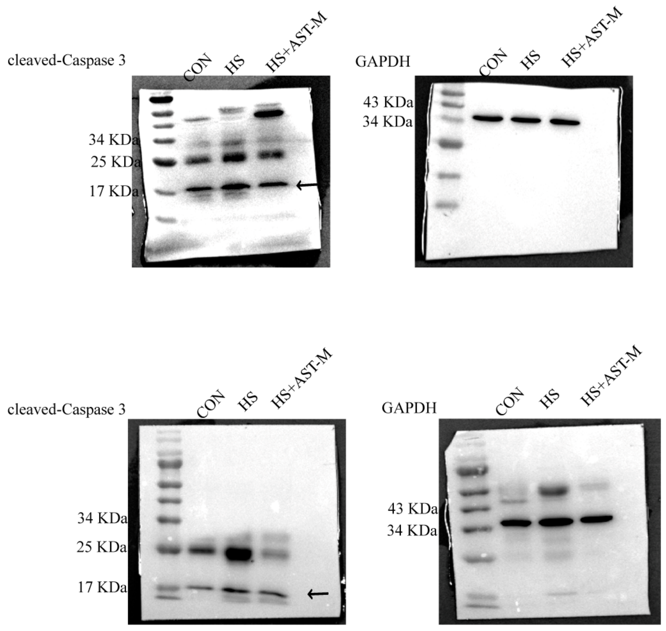

https://doi.org/10.3390/xxxxx

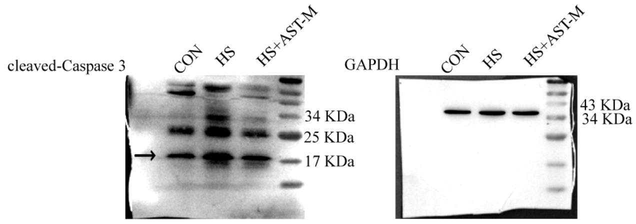

	cleaved-Caspase 3	GAPDH	Gray ratio	Data normaliza-tion
CON	31129.765	44862.986	0.693885267	1.089893079
HS	45897.421	43015.208	1.067004512	1.675955505
HS+IDE-M	27198.078	48896.3	0.556240002	0.873692175
CON	22019.3	42927.359	0.512943272	0.805685535
HS	44789.886	52012.622	0.861134938	1.352593942
HS+IDE-M	31002.957	47895.108	0.647309471	1.016735973
CON	30087.057	42789.886	0.703134778	1.104421386
HS	51981.957	45601.401	1.139920175	1.790484924
HS+IDE-M	39013.836	44586.451	0.875015506	1.374396302
